# Beyond Cause of Death: Translating Medico-Legal Autopsy Evidence into Risk Mitigation, Organizational Learning, and Institutional Adoption

**DOI:** 10.3390/diagnostics16213611

**Published:** 2026-11-01

**Authors:** Živana Slović, Snežana Knežević, Stefan Milojević

**Affiliations:** 1 Department of Forensic Medicine, Faculty of Medical Sciences, University of Kragujevac, Svetozara Markovića 69, 34000 Kragujevac, Serbia; zivanaminic@yahoo.com; 2 Department of Forensic Medicine and Toxicology, University Clinical Center Kragujevac, 34000 Kragujevac, Serbia; 3 Center for Neurosciences, Faculty of Medical Sciences, University of Kragujevac, Svetozara Markovića 69, 34000 Kragujevac, Serbia; 4 Faculty of Organizational Sciences, University of Belgrade, 11000 Belgrade, Serbia; snezana.knezevic@fon.bg.ac.rs; 5 Faculty of Medical Sciences, University of Kragujevac, Svetozara Markovića 69, 34000 Kragujevac, Serbia; 6 Faculty of Business Economics, Educons University, 21208 Sremska Kamenica, Serbia

**Keywords:** medico-legal autopsy, postmortem evidence, diagnostic discrepancies, healthcare risk management, organizational learning, perceived system value, institutional adoption

## Abstract

**Background/Objectives:** Although medico-legal autopsy remains an important source of postmortem evidence for clarifying causes and circumstances of death, identifying diagnostic discrepancies, and evaluating adverse clinical outcomes, its relevance may extend well beyond individual case resolution. Evidence generated after death can contribute to healthcare risk management, organizational learning, and institutional decision-making, yet the mechanisms through which these benefits acquire broader system value remain insufficiently understood. This study therefore examined relationships among medico-legal risk mitigation, financial risk mitigation, organizational learning capability, perceived system value, and institutional adoption intention. **Methods:** A cross-sectional survey of 1035 professionals working in healthcare, forensic medicine, pathology, legal, and administrative settings in Serbia was analyzed using exploratory and confirmatory factor analyses, structural equation modeling, and bootstrap mediation analysis. **Results:** Greater medico-legal risk mitigation was associated with both financial risk mitigation and perceived system value, whereas financial risk mitigation and organizational learning capability independently contributed to perceived system value. In turn, perceived system value showed the strongest direct relationship with institutional adoption intention. Mediation analyses further identified an indirect learning pathway and a sequential mechanism linking medico-legal risk mitigation with institutional adoption through financial risk mitigation and perceived system value. **Conclusions:** These findings position postmortem evidence as a potential source of diagnostic and organizational intelligence rather than solely as the endpoint of retrospective investigation. Its broader institutional relevance depends on the extent to which evidence generated through autopsy can be translated into risk mitigation, organizational learning, and recognized system value that supports systematic integration into healthcare practice.

## 1. Introduction

Adverse clinical outcomes can generate consequences extending beyond the immediate episode of care, including concerns related to patient safety, professional liability, financial exposure, organizational accountability, and quality of care [1,2]. Medico-legal autopsy can contribute to managing these consequences by revealing diagnostic discrepancies, clarifying relationships between clinical interventions and fatal outcomes, providing objective evidence in disputed cases, and supporting quality assessment and professional learning [3,4,5]. Contemporary evidence confirms that clinically important diagnoses missed before death remain detectable at autopsy despite advances in diagnostic technologies [6,7]. Yet autopsy utilization remains constrained by barriers related to consent and counseling practices, as well as clinician perspectives toward autopsy [8]. This creates an institutional paradox: autopsy continues to generate clinically and organizationally relevant information [9], but demonstrated usefulness does not necessarily translate into sustained institutional use [10,11]. Existing research has examined diagnostic discrepancies, quality assurance, education, malpractice, costs, and barriers to autopsy utilization, but these domains have largely been considered separately [12,13]. Consequently, it remains unclear how the diagnostic and medico-legal information generated through autopsy is associated with perceived financial and organizational-learning benefits, broader system value, and institutional willingness to adopt its systematic use.

The present study addresses this gap by shifting attention from the established diagnostic and evidentiary utility of medico-legal autopsy to how that utility becomes organizationally consequential. Its novelty lies in integrating Medico-Legal Risk Mitigation, Financial Risk Mitigation, Organizational Learning Capability, Perceived System Value, and Institutional Adoption Intention within a single framework of hypothesized associations. Rather than assuming a direct association between recognized benefits and institutional support, the study conceptualizes Perceived System Value as an evaluative construct potentially linking specific perceived benefits with an organizational rationale for adoption. Accordingly, the study addresses the following research question (RQ): How are medico-legal risk mitigation, financial risk mitigation, and organizational learning capability associated with perceived system value and institutional adoption intention in the context of medico-legal autopsy?

Medico-legal clarification may be relevant to reducing uncertainty surrounding professional liability, malpractice claims, administrative investigations, litigation, and institutional responsibility [14,15]. Its organizational significance may therefore extend beyond legal clarification to financial risk mitigation, given the potential relevance of reliable evidence to exposure associated with disputes, claims, and unnecessary procedures [16]. This is particularly relevant because the direct costs of autopsy, including specialized personnel, infrastructure, and laboratory procedures, are immediately visible, whereas its potential economic benefits are indirect and dispersed over time [17,18]. From a healthcare-system perspective, the relevant issue is therefore not simply what autopsy costs, but whether its perceived risk-mitigation benefits are associated with sufficient system value and institutional support for adoption [19].

Autopsy can also contribute to organizational learning by enabling healthcare organizations to compare clinical assumptions made during life with evidence obtained after death [20,21]. Diagnostic discrepancies, unrecognized conditions, complications, and procedural weaknesses identified postmortem can inform professional learning and protocol improvement [22,23]. The potential organizational relevance of such information is associated with its interpretation, dissemination, incorporation into institutional memory, and consideration in future practices and decisions. Autopsy is therefore retrospective in the event it investigates but potentially prospective in its organizational function.

Perceived System Value consequently provides the conceptual bridge between benefits identified at the case level and institutional action. Using data from a multidisciplinary sample of professionals in Serbia, the study tests an integrated structural model incorporating direct, mediating, and sequential relationships among these dimensions. By connecting the evidentiary role of medico-legal autopsy with risk mitigation, financial consequences, organizational learning, system value, and institutional adoption, the study positions postmortem evidence at the intersection of diagnostic evaluation, healthcare risk management, and institutional decision-making. Its contribution lies in examining how evidence generated after an adverse clinical outcome is associated with perceived organizational value for learning, risk management, and future healthcare decision-making, beyond its established role in retrospective case clarification.

## 2. Literature Review and Hypothesis Development

### 2.1. Medico-Legal Autopsy as a Mechanism of Risk Mitigation

Although medico-legal autopsy is conventionally associated with establishing cause and manner of death and resolving diagnostic uncertainty, its relevance extends beyond the resolution of an individual case [24]. Autopsy findings can clarify discrepancies between clinical and postmortem diagnoses, document previously unidentified conditions, and provide evidence capable of resolving uncertainty surrounding adverse outcomes. The continuing diagnostic relevance of autopsy is well established. Shojania et al. [24], in their systematic review of autopsy-detected diagnostic errors, demonstrated that clinically important discrepancies persist despite advances in diagnostic technology. Earlier work by Hill and Anderson [25] similarly positioned systematic comparison between clinical and autopsy diagnoses as a valuable component of quality assessment and improvement.

The organizational significance of such evidence can be understood through Reason’s [26] systems approach to human error. Rather than attributing adverse outcomes exclusively to individual actions, the systems perspective recognizes that errors may reflect latent weaknesses embedded within organizational processes and safeguards [27]. Postmortem investigation is particularly relevant in this context because it can reveal information that was unavailable, overlooked, or incorrectly interpreted before death. Autopsy can therefore function not merely as retrospective confirmation of an outcome, but as a source of postmortem evidence capable of identifying medico-legal vulnerabilities associated with diagnostic uncertainty, documentation, accountability, and potential disputes [28].

These medico-legal vulnerabilities can also generate financial consequences. Unresolved uncertainty surrounding adverse outcomes may expose healthcare organizations to litigation, compensation claims, additional investigation, administrative burden, and other costs [29,30]. Accordingly, medico-legal and financial consequences should not necessarily be conceptualized as independent domains. Greater capacity to clarify the circumstances surrounding an adverse outcome may be associated with lower perceived uncertainty and financial exposure [31,32]. This reasoning leads to the first hypothesis:

**H1.**  *Perceived medico-legal risk mitigation is positively associated with perceived financial risk mitigation associated with medico-legal autopsy.*

Medico-legal risk reduction may also be associated with professionals’ broader evaluation of autopsy [33,34]. From a systems perspective, evidence acquires organizational significance when it enables institutions to identify and respond to vulnerabilities that extend beyond the individual event [26]. By improving evidentiary clarity, strengthening accountability, and reducing medico-legal uncertainty, autopsy may therefore be perceived as contributing to healthcare governance beyond its immediate forensic purpose [35,36]. This shifts its evaluation from procedural utility toward system-level value. When postmortem investigation is perceived as strengthening institutional capacity to understand and manage medico-legal vulnerability, its relevance should extend to how professionals evaluate its contribution to the healthcare system as a whole [37]. Accordingly:

**H2.**  *Perceived medico-legal risk mitigation is positively associated with the perceived system value of medico-legal autopsy.*

### 2.2. Financial Risk Mitigation and Perceived System Value

Evaluation of the institutional value of medico-legal autopsy should not be reduced to the direct cost of performing the procedure [38]. Such an approach overlooks the potential value of information generated through postmortem investigation. The perceived value of an organizational practice may reflect not only its immediate benefits but also its potential relevance to preventing or reducing future losses [39]. In the context of autopsy, its economic relevance may therefore lie partly in the downstream organizational consequences that can be avoided when postmortem evidence is systematically incorporated into risk management and quality assurance [40].

This perspective is consistent with the broader systems approach to healthcare safety, which directs attention toward organizational conditions and the prevention of recurrent adverse outcomes rather than isolated individual errors [26]. Autopsy-based quality-assurance models likewise emphasize that systematic analysis of diagnostic discrepancies can contribute to improvements in medical care rather than merely documenting failure after the event [25]. Financial Risk Mitigation can therefore form part of a broader assessment of institutional usefulness. When professionals perceive autopsy findings as capable of limiting downstream exposure and avoidable organizational costs, the procedure may be evaluated less as an isolated expenditure and more as an investment in institutional risk management and quality assurance [41,42]. Thus:

**H3.**  *Perceived financial risk mitigation is positively associated with the perceived system value of medico-legal autopsy.*

### 2.3. Organizational Learning Capability and the System Value of Autopsy

The institutional usefulness of information depends not only on its availability but also on an organization’s ability to learn from it [43]. Organizational learning theory distinguishes information acquisition from the processes through which information modifies organizational knowledge and action. Argyris and Schön [44] conceptualized organizational learning as occurring when organizations detect and respond to discrepancies in ways that affect their theories of action. This distinction is especially relevant to postmortem evidence: discovering a discrepancy is not equivalent to learning from it [45,46]. High-reliability theory provides a complementary explanation. Organizations operating in high-risk environments sustain reliability partly by maintaining sensitivity to failure and treating errors, anomalies, and unexpected events as opportunities for learning rather than isolated exceptions [47,48]. Adverse outcomes can therefore contain information about weaknesses that remains organizationally relevant after the immediate event has ended [49].

Autopsy is particularly compatible with this logic. Comparison of premortem and postmortem findings can reveal diagnostic discrepancies that would otherwise remain unknown, while systematic analysis of these discrepancies has long been proposed as a mechanism for quality assurance [24,25]. The organizational significance of such evidence, however, depends on whether healthcare institutions can interpret findings, communicate lessons, revise procedures, and prevent recurrence [50]. Organizational Learning Capability therefore captures the capacity to interpret and utilize autopsy-derived evidence as knowledge relevant to future action. Higher perceived Organizational Learning Capability is expected to be associated with greater perceived system value of medico-legal autopsy [51]. Thus:

**H4.**  *Organizational learning capability derived from medico-legal autopsy is positively associated with its perceived system value.*

### 2.4. Perceived System Value and Institutional Adoption

Recognition of individual benefits does not necessarily imply institutional commitment [52]. For a practice to become embedded within organizational routines, decision-makers and professionals must perceive that its advantages are sufficiently meaningful at the system level to justify continued or expanded use. This distinction can be grounded in Rogers’ [53] Diffusion of Innovations framework, which emphasizes the relevance of perceived innovation attributes—particularly relative advantage—to adoption decisions. Although medico-legal autopsy is not a technological innovation, decisions concerning its systematic institutional utilization involve an analogous evaluative process [54]. Healthcare organizations must determine whether the knowledge and risk-related benefits generated through autopsy justify allocating resources, establishing supporting procedures, and incorporating its findings into organizational routines [55]. Perceived System Value captures this broader assessment by locating the usefulness of autopsy beyond the outcome of an individual examination. This distinction is particularly relevant given the historical decline in autopsy utilization despite continuing evidence of clinically important diagnostic discrepancies [56]. The diagnostic usefulness of postmortem evidence alone does not guarantee its systematic institutional use. Rather, such evidence must also be recognized as consequential for the wider healthcare system before it can provide a rationale for institutional commitment [57]. Accordingly:

**H5.**  *Perceived system value is positively associated with institutional adoption intention toward medico-legal autopsy.*

### 2.5. Financial Risk Mitigation as a Mediating Mechanism

The preceding arguments establish that medico-legal and financial risk mitigation represent related but conceptually distinct sources of institutional value [58]. Their combination also suggests a potential indirect association. Medico-legal clarification may be associated with broader perceived system value through its relationship with perceived protection against financial and organizational exposure [59]. Financial Risk Mitigation is therefore conceptualized as an intermediate variable in the hypothesized indirect association between Medico-Legal Risk Mitigation and Perceived System Value. Importantly, this proposition does not merely reproduce the direct associations proposed in H1 and H3; it examines whether Medico-Legal Risk Mitigation is indirectly associated with Perceived System Value through Financial Risk Mitigation [60,61]. Accordingly:

**H6.**  *Financial risk mitigation statistically mediates the positive association between medico-legal risk mitigation and the perceived system value of medico-legal autopsy.*

### 2.6. Perceived System Value as a Bridge Between Learning and Adoption

The distinction between organizational learning and institutional adoption raises a different mediation question [62,63]. An organization may possess the capability to derive lessons from autopsy findings without necessarily demonstrating a corresponding intention to support the continued use of autopsy. An important consideration is whether perceived organizational learning capability is associated with broader perceived system value [64]. Perceived System Value is therefore proposed as an intermediate construct in the association between Organizational Learning Capability and Institutional Adoption Intention. Rather than assuming a direct correspondence between learning capability and institutional commitment, the model examines whether Organizational Learning Capability is indirectly associated with Institutional Adoption Intention through Perceived System Value. This provides a conceptual bridge between the organizational capability described by Argyris and Schön [44] and high-reliability theory [47,48] and the adoption logic associated with perceived advantage in Rogers’ [53] framework. Accordingly:

**H7.**  *Perceived system value statistically mediates the positive association between organizational learning capability and institutional adoption intention toward medico-legal autopsy.*

### 2.7. Sequential Value Translation and Institutional Adoption

The final hypothesis integrates the risk-related associations into a single sequential statistical model. The proposed sequence represents a theoretically specified pattern of associations among organizational domains: perceived medico-legal risk mitigation is associated with perceived financial risk mitigation, which is associated with Perceived System Value, which, in turn, is associated with Institutional Adoption Intention [65]. This sequential proposition differs conceptually from the preceding mediation hypotheses. H6 examines the indirect association between Medico-Legal Risk Mitigation and Perceived System Value through Financial Risk Mitigation, whereas H8 extends this statistical specification to Institutional Adoption Intention [66,67]. The sequence therefore represents a hypothesized pattern of indirect associations rather than an empirically established temporal or causal process. This integration also connects Reason’s [26] systems perspective with Rogers’ [53] adoption logic. The former highlights the potential organizational relevance of information concerning institutional vulnerabilities, while the latter emphasizes the relevance of perceived advantages to adoption decisions. The sequential model brings these perspectives together by examining whether case-related medico-legal benefits are indirectly associated with Institutional Adoption Intention through perceived financial risk mitigation and system value [68]. Accordingly:

**H8.**  *Financial risk mitigation and perceived system value sequentially statistically mediate the positive association between medico-legal risk mitigation and institutional adoption intention toward medico-legal autopsy.*

**Figure 1:**
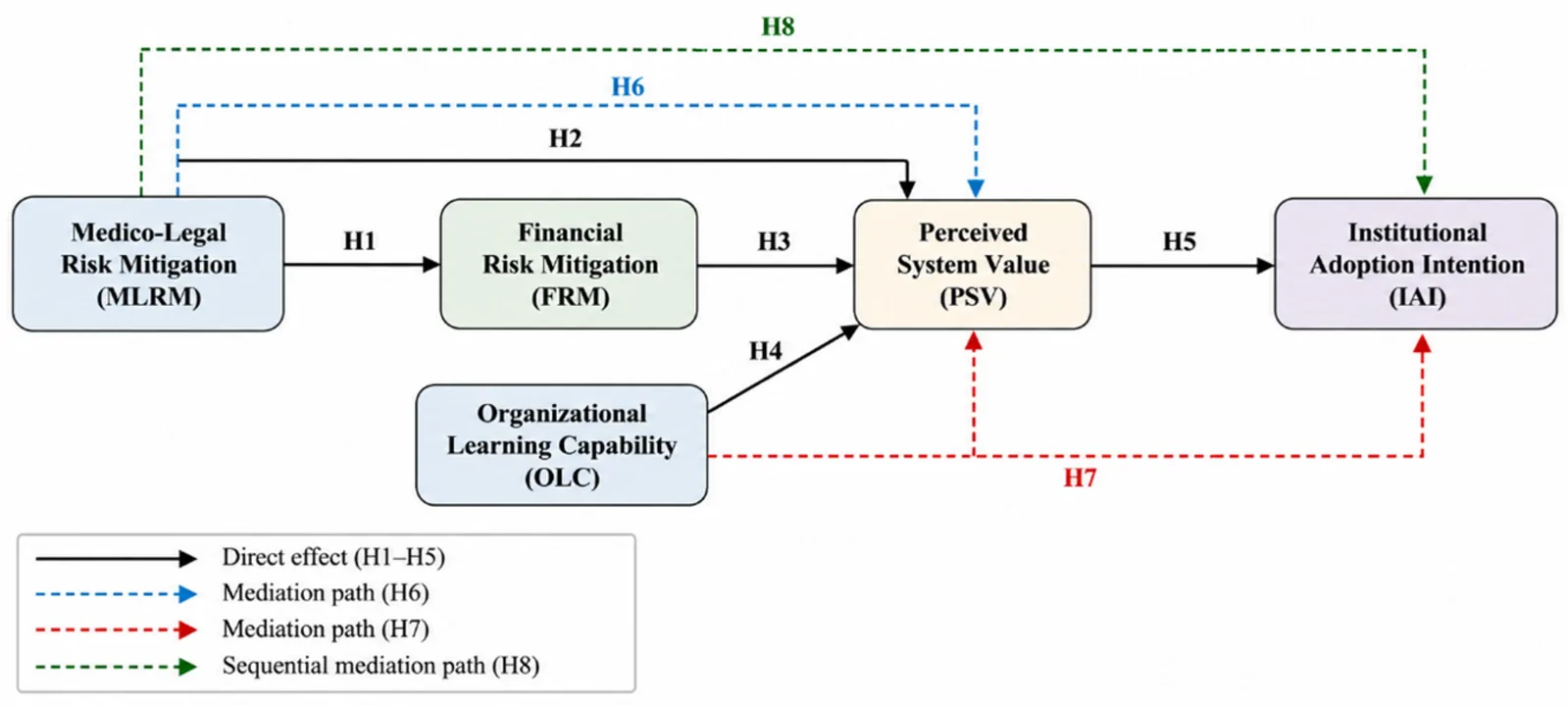
Conceptual Model and Hypothesized Relationships.

The proposed conceptual model, integrating the direct associations and hypothesized indirect and sequential statistical relationships specified in H1–H8, is presented in Figure 1.

## 3. Materials and Methods

### 3.1. Participants and Data Collection

The study was conducted in Serbia between May and September 2026 among professionals working in healthcare, medico-legal, legal, and administrative settings. Eligible participants were professionals aged 18 years or older who were employed in healthcare, forensic medicine, pathology, legal services, healthcare management, or related institutional administration, and whose professional responsibilities or expertise were relevant to medico-legal autopsy procedures, their findings, or their organizational, legal, and financial implications. Direct involvement in performing medico-legal autopsies was not required, as the study also included professionals engaged in healthcare governance, institutional risk management, and legal or administrative decision-making. Participation was voluntary. Data collection was organized through direct institutional contact. The authors personally approached the participating institutions and established contact with the personnel responsible for facilitating research access. Following institutional coordination, the designated contact persons distributed the questionnaire electronically via email to eligible employees within their respective organizations.

A total of 1658 eligible professionals were invited to participate in the study through the 15 participating institutions. Of these, 1220 returned questionnaires, corresponding to a survey response rate of 73.6%. Following data screening, 185 questionnaires were excluded because of incomplete responses, resulting in a final analytical sample of 1035 participants. The questionnaire usability rate was 84.8% of returned questionnaires, while the final analytical sample represented 62.4% of all invited professionals. The sample was nearly evenly distributed by gender, with 50.6% male and 49.4% female participants. Most respondents were established professionals: 29.6% were aged 36–45 years and 30.6% were aged 46–55 years, while 22.8% were 35 years or younger and 17.0% were aged 56 years or older. Clinicians constituted the largest professional group (39.7%), followed by healthcare managers or administrators (18.0%), legal professionals (17.5%), forensic medicine specialists (14.0%), and pathology specialists (10.8%). The sample was characterized by substantial professional experience, with 66.6% of respondents reporting more than 10 years of professional practice. Importantly, 63.4% had direct professional experience with medico-legal autopsies or their findings, indicating substantial practical exposure to the subject under investigation.

More than half of the participants worked primarily in public healthcare institutions (54.8%), while the remainder represented judicial or public institutions (16.2%), private healthcare institutions (15.4%), and university or research institutions (13.6%). Overall, the sample reflects a professionally experienced and institutionally diverse group with considerable direct exposure to medico-legal practice.

### 3.2. Questionnaire Development and Measurement

The questionnaire was developed specifically for the present study using a literature-informed and theory-driven approach. Because no existing instrument comprehensively captured the medico-legal, organizational, financial, and institutional implications of medico-legal autopsy relevant to the objectives of this study, an initial pool of 30 items (v1–v30) was formulated. The items were developed to represent different potential consequences and uses of medico-legal autopsy identified in the relevant literature, without imposing an a priori empirical factor structure. All items were measured using a five-point Likert scale ranging from 1 (strongly disagree) to 5 (strongly agree).

The content of the initial item pool was informed first by the established literature on the diagnostic and quality-assurance functions of autopsy. Previous research has demonstrated that postmortem examination can identify clinically important discrepancies between premortem and postmortem diagnoses and provide information that would otherwise remain unavailable after a patient’s death [24,25]. This literature informed the formulation of items addressing factual clarification, uncertainty surrounding cause and circumstances of death, diagnostic discrepancies, professional liability, transparency, disputed cases, and the potential use of postmortem findings for improving healthcare practice. A second conceptual foundation was provided by the systems approach to error and organizational risk. Reason [26] argues that adverse outcomes should not be understood exclusively in terms of individual error because they may reveal latent weaknesses embedded within organizational systems. This perspective informed items addressing the capacity of postmortem findings to identify systemic weaknesses and clarify potential professional and institutional exposure. It also supported the inclusion of items concerning the wider consequences of unresolved medico-legal uncertainty, including legal and administrative costs, financial exposure, risk assessment, and prevention of future organizational losses.

The formulation of items concerning the use of autopsy-derived information for improvement was informed by organizational learning theory. Argyris and Schön [44] distinguish the availability of information from organizational learning that occurs when information is incorporated into organizational knowledge and action. Accordingly, the initial questionnaire included items addressing the identification of previously unrecognized problems, learning from previous clinical cases, professional learning, revision and improvement of clinical protocols, and prevention of recurrence. High-reliability theory provided an additional theoretical basis for these items. High-reliability organizations maintain sensitivity to failures and unexpected outcomes and use them as sources of information about vulnerabilities within complex systems [47,48]. Applied to medico-legal autopsy, this perspective supported the formulation of items examining whether information generated retrospectively after death could be used prospectively to identify weaknesses, improve procedures, strengthen risk management, and reduce the likelihood or consequences of similar future events.

The initial item pool also addressed respondents’ evaluations of the broader organizational usefulness of medico-legal autopsy. Items were therefore formulated to assess whether the information and benefits generated through autopsy were perceived as justifying the resources required for its performance, outweighing immediate costs, contributing to more efficient resource use, supporting risk management, and warranting long-term investment. The conceptual basis for incorporating such evaluative judgments was consistent with Rogers’ [53] Diffusion of Innovations framework, particularly the concept of perceived relative advantage. Rogers emphasizes that adoption is influenced not simply by the existence of potential benefits, but by the extent to which those benefits are perceived as advantageous by potential adopters. Finally, the questionnaire included items addressing the institutional implications of these perceived benefits. These items concerned the systematic use of autopsy findings in risk management, healthcare quality and safety decisions, allocation of resources for medico-legal autopsy capacity, incorporation into institutional strategies, and support for broader institutional use. The distinction between perceived usefulness and willingness to institutionalize a practice was again informed by Rogers’ [53] adoption framework, allowing the questionnaire to capture not only respondents’ evaluations of medico-legal autopsy but also their support for its integration into organizational practice.

Prior to the main data collection, the initial 30-item questionnaire underwent an expert review conducted by three subject-matter experts. The review focused on the relevance of the proposed items to the study objectives, their conceptual appropriateness, clarity of wording, and coverage of the medico-legal, organizational, financial, and institutional dimensions of the research topic. This procedure provided a preliminary qualitative assessment of content validity before the instrument was administered to the study participants. Subsequent empirical validation was performed through exploratory and confirmatory factor analyses, as described in the following sections.

Thus, the initial questionnaire comprised 30 literature-informed items covering complementary aspects of the potential institutional relevance of medico-legal autopsy. Importantly, the empirical dimensionality of these items was not assumed in advance. Instead, the underlying structure was subsequently examined through exploratory factor analysis (EFA). Item retention was based on the magnitude of primary factor loadings, with 0.50 established as the minimum acceptable threshold. Five items (v1, v8, v13, v20, and v26) were removed because they did not meet this criterion, resulting in a final set of 25 items for subsequent measurement and structural analyses. The complete initial pool of 30 items, together with the retention status of each item, is provided in Appendix A (Table A1).

### 3.3. Data Analysis

The data were analyzed using IBM SPSS Statistics (version 28; IBM Corp., Armonk, NY, USA) for data screening, descriptive statistics, exploratory factor analysis (EFA), and full-collinearity diagnostics, and IBM SPSS Amos (version 28; IBM Corp., Armonk, NY, USA) for confirmatory factor analysis (CFA) and structural equation modeling (SEM), including bootstrap mediation analyses. The dimensional structure of the initial 30-item questionnaire was examined using exploratory factor analysis (EFA) with Maximum Likelihood (ML) extraction. Although the items were measured on a five-point Likert scale and are therefore ordinal, ML extraction was applied as a continuous-variable approximation. This analytical choice was considered in light of the five response categories and the relatively large sample size (N = 1035). However, the use of ML does not eliminate potential sensitivity to ordinal measurement characteristics or departures from multivariate normality. To assess the robustness of the factor structure to the choice of extraction method, an additional exploratory factor analysis was performed using Principal Axis Factoring (PAF). The PAF analysis reproduced the same five-factor structure and retained the same 25 items as the original ML solution, indicating that the identified dimensional structure was stable across the two extraction methods. The suitability of the data for factor analysis was assessed using the Kaiser–Meyer–Olkin (KMO) measure and Bartlett’s test of sphericity.

Factor retention was guided by eigenvalues greater than 1 and the interpretability of the extracted solution. Item retention required a primary factor loading of at least 0.50. Accordingly, five items (v1, v8, v13, v20, and v26) were excluded, leaving 25 items for subsequent analyses. The retained items were also examined for cross-loadings to assess whether they demonstrated a distinguishable association with their respective factors. The final rotated factor solution showed no substantial cross-loadings, as reported in Table 1.

Potential common method bias was initially assessed using Harman’s single-factor test, with the variance explained by the first unrotated factor evaluated against the 50% criterion. To provide an additional diagnostic assessment, full-collinearity testing was conducted using composite construct scores, following the approach proposed by Kock [69]. Composite scores were calculated as the mean of the five retained items for each construct. Each of the five constructs was subsequently regressed on the remaining four constructs, and full-collinearity variance inflation factors (VIFs) were calculated as 1/(1 − R^2^). A threshold of 3.3 was applied to identify potentially problematic full collinearity. The detailed results are reported in Appendix B (Table A2).

The resulting measurement structure was evaluated through confirmatory factor analysis (CFA). Construct reliability and convergent validity were assessed using composite reliability (CR) and average variance extracted (AVE), applying recommended thresholds of 0.70 and 0.50, respectively. Discriminant validity was examined using both the Fornell–Larcker criterion and the heterotrait–monotrait ratio of correlations (HTMT), with HTMT values below 0.85 considered indicative of adequate discriminant validity. Standardized residual covariances were also examined to identify potential localized model misspecification, using an absolute value of 2.58 as a diagnostic threshold.

The hypothesized relationships were tested using structural equation modeling (SEM). Model adequacy was evaluated using χ^2^, χ^2^/df, CFI, TLI, GFI, and RMSEA with its 90% confidence interval and PCLOSE. Direct relationships corresponding to H1–H5 were evaluated using standardized regression coefficients (β), together with their standard errors, critical ratios, and significance levels. The indirect effects specified in H6–H8 were examined using 5000 bootstrap resamples and 95% percentile confidence intervals, with an indirect effect considered significant when its confidence interval did not include zero. This procedure included both single-mediator indirect associations and the hypothesized sequential indirect association. The mediation coefficients were estimated as unstandardized indirect effects. The explanatory capacity of the structural model was evaluated through R^2^ values for the endogenous constructs.

## 4. Results

The data were suitable for factor analysis, as indicated by the excellent KMO value (0.953) and a significant Bartlett’s test of sphericity, χ^2^(435) = 17,239.990, *p* < 0.001. Maximum Likelihood extraction identified five factors with eigenvalues greater than 1, which together explained 57.36% of the total variance, supporting a five-factor structure. Harman’s single-factor test showed that the first factor explained 30.63% of the total variance, below the conventional 50% threshold. Given the limitations of this test, an additional full-collinearity assessment was conducted using composite construct scores, following Kock [69]. The resulting VIF values ranged from 1.255 to 2.183, remaining below the recommended threshold of 3.3. These findings provided supplementary evidence against substantial common method concerns, although common method bias cannot be definitively excluded. Detailed results are presented in Appendix B (Table A2).

As shown in Table 1, the exploratory factor analysis supported a clear five-factor structure, with all items loading strongly on their intended factors and no substantial cross-loadings. Factor loadings ranged from 0.635 to 0.889, providing support for the factorial structure of the measurement instrument.

As shown in Table 2, all constructs demonstrated satisfactory reliability and convergent validity, with CR values ranging from 0.872 to 0.893 and AVE values from 0.578 to 0.628, exceeding the recommended thresholds of 0.70 and 0.50, respectively.

Discriminant validity was supported by the Fornell–Larcker criterion (Table 3), as the square root of AVE for each construct exceeded its correlations with the remaining constructs. The HTMT ratios (Table 4) ranged from 0.079 to 0.680 and remained below the recommended threshold of 0.85, providing additional evidence of discriminant validity. The interconstruct correlations ranged from 0.084 to 0.675 (Table 3), indicating that the five constructs were related but empirically distinguishable. Additional residual diagnostics were conducted to assess potential localized model misspecification, particularly given the semantic proximity of certain questionnaire items. Examination of the standardized residual covariance matrix showed that all 300 unique off-diagonal residuals fell within the ±2.58 threshold. Standardized residuals ranged from −1.984 to 1.882, with no values exceeding the specified threshold. These results indicated no pronounced localized discrepancies between the observed and model-implied covariance structures, providing further support for the adequacy of the five-factor measurement model.

**Figure 2:**
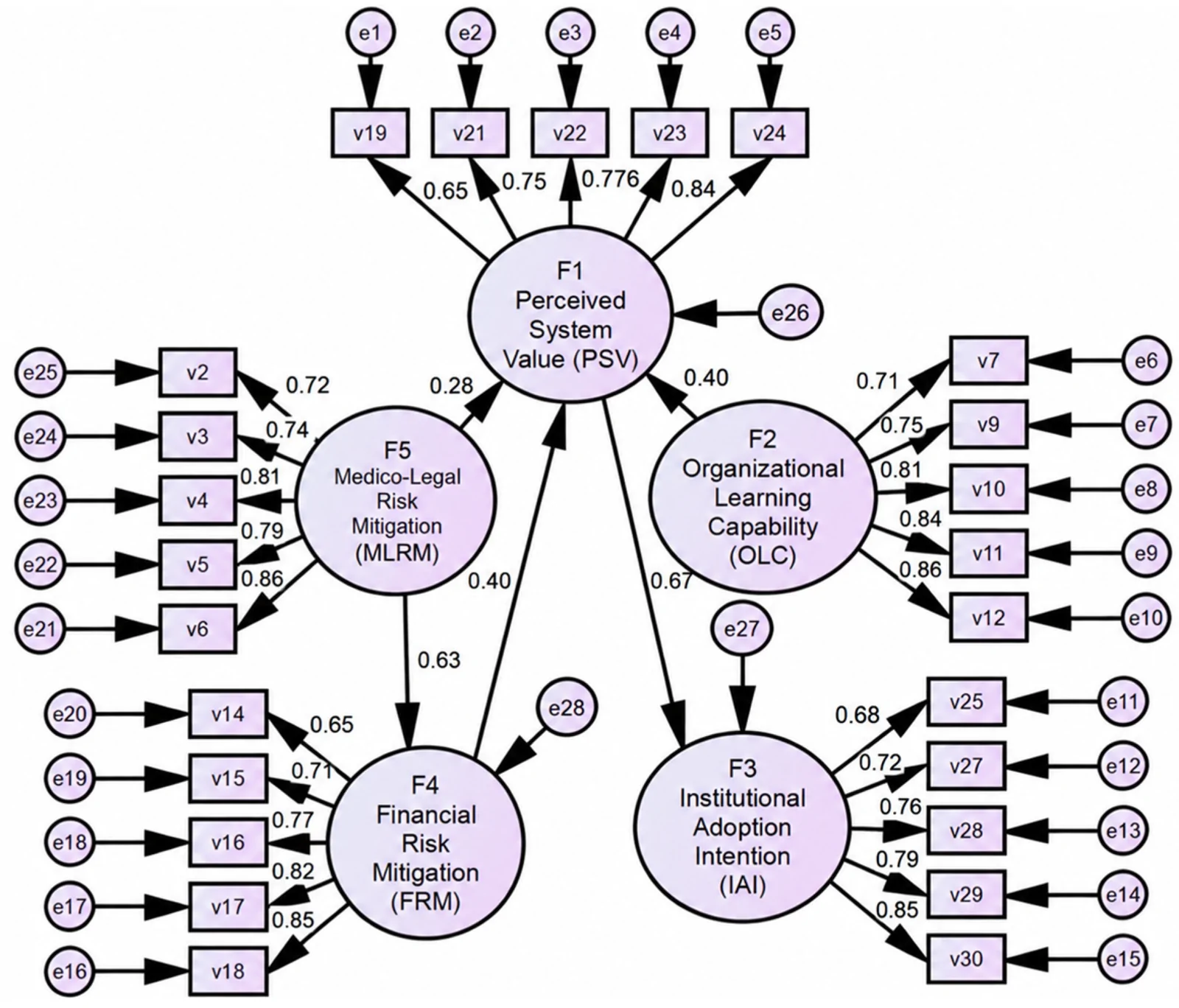
Structural Equation Model of Medico-Legal Autopsy Value, Risk Mitigation, Organizational Learning, and Institutional Adoption.

The structural model demonstrated a good fit to the data, χ^2^(270) = 307.543, *p* = 0.058, χ^2^/df = 1.139, CFI = 0.997, TLI = 0.997, GFI = 0.977, and RMSEA = 0.012 (90% CI [0.000, 0.017], PCLOSE = 1.000), indicating that the specified model adequately reproduced the observed covariance structure. Figure 2 presents the proposed structural model and the relationships among the five constructs. Medico-Legal Risk Mitigation is positively associated with both Financial Risk Mitigation and Perceived System Value. Financial Risk Mitigation and Organizational Learning Capability are also positively associated with Perceived System Value. Perceived System Value, in turn, is positively associated with Institutional Adoption Intention, highlighting its central position within the proposed structural framework.

The direct structural relationships are reported in Table 5. Medico-Legal Risk Mitigation was significantly and positively associated with Financial Risk Mitigation (β = 0.629, *p* < 0.001) and Perceived System Value (β = 0.284, *p* < 0.001), supporting H1 and H2. Financial Risk Mitigation was also positively associated with Perceived System Value (β = 0.401, *p* < 0.001), supporting H3. A similarly positive association with Perceived System Value was identified for Organizational Learning Capability (β = 0.402, *p* < 0.001), supporting H4. Finally, Perceived System Value exhibited the largest standardized direct association in the model with Institutional Adoption Intention (β = 0.674, *p* < 0.001), supporting H5. Consequently, all five direct hypotheses were supported.

The results of the statistical mediation analysis are presented in Table 6. A significant indirect association was identified between Medico-Legal Risk Mitigation and Perceived System Value through Financial Risk Mitigation (indirect effect = 0.191, 95% CI [0.155, 0.230], *p* < 0.001), supporting H6. A significant indirect association was also observed between Organizational Learning Capability and Institutional Adoption Intention through Perceived System Value (indirect effect = 0.255, 95% CI [0.213, 0.301], *p* < 0.001), supporting H7. The hypothesized sequential indirect association linking Medico-Legal Risk Mitigation, Financial Risk Mitigation, Perceived System Value, and Institutional Adoption Intention was also statistically significant (indirect effect = 0.132, 95% CI [0.105, 0.161], *p* < 0.001), supporting H8. None of the bootstrap confidence intervals included zero, providing statistical support for all hypothesized indirect associations without establishing causal mediation or temporal ordering.

The structural model accounted for 39.6% of the variance in Financial Risk Mitigation, 54.6% in Perceived System Value, and 45.5% in Institutional Adoption Intention (Table 7). The highest proportion of accounted-for variance was observed for Perceived System Value, with the specified predictors jointly accounting for more than half of its variance. Overall, the R^2^ values indicate substantial proportions of variance accounted for across the endogenous constructs.

## 5. Discussion

The present study extends the discussion of medico-legal autopsy beyond its established diagnostic, forensic, and evidentiary functions by examining how these functions are associated with broader perceived institutional value. The findings identify a structured pattern of associations linking the perceived benefits of postmortem information with its potential relevance to the functioning of healthcare organizations. This interpretation extends the established understanding of autopsy as a mechanism for detecting discrepancies between premortem diagnoses and postmortem findings. The landmark systematic review by Shojania et al. [24] demonstrated that autopsy continues to reveal clinically important diagnostic errors despite advances in diagnostic technology. The present findings add an organizational dimension to this established diagnostic role: the significance of information generated through autopsy may lie not only in identifying what was missed in an individual case, but also in its capacity to inform how institutions recognize risk, learn from failure, evaluate system-level value, and consider the institutional use of autopsy.

### 5.1. From Medico-Legal Evidence to Organizational Risk Intelligence

The strong relationship between Medico-Legal Risk Mitigation and Financial Risk Mitigation provides an important starting point for interpreting the model. It suggests that respondents perceive medico-legal risk mitigation and financial risk mitigation as closely related dimensions of autopsy value. The perceived value of autopsy may extend beyond the immediate medico-legal case, particularly in relation to its potential relevance for clarifying causation, documenting findings, resolving uncertainty, and identifying potential failures. Such information may also be relevant to liability exposure, dispute resolution, resource loss, and the organizational costs associated with unresolved or repeated failures. This finding is particularly meaningful when viewed through Reason’s [70] organizational accident perspective. In complex systems, adverse outcomes rarely result from a single isolated error; rather, they emerge through combinations of active failures and latent organizational conditions. From this perspective, medico-legal autopsy can be understood as a retrospective window into conditions that may otherwise remain concealed. Its institutional importance therefore does not depend solely on establishing cause of death or documenting diagnostic discrepancies. Postmortem findings can also expose vulnerabilities whose consequences extend across clinical, legal, managerial, and financial domains.

The statistical mediation result is consistent with this interpretation. A significant indirect association was identified between Medico-Legal Risk Mitigation and Perceived System Value through Financial Risk Mitigation. This finding suggests that perceived medico-legal usefulness is associated with broader perceived institutional value partly through its relationship with perceived financial risk mitigation, without establishing a causal transmission mechanism. The value attributed to autopsy is therefore not merely intrinsic to the procedure; it also reflects the extent to which medico-legal clarification is perceived to reduce consequential financial and organizational exposure.

### 5.2. Autopsy as an Organizational Learning Infrastructure

A second and conceptually distinct finding concerns Organizational Learning Capability. Its positive association with Perceived System Value suggests that the perceived institutional value of medico-legal autopsy is related not only to the information produced but also to professionals’ perceptions of organizational capacity to utilize that information for learning. This distinction is central to contemporary thinking about learning healthcare organizations. Learning requires more than the availability of information; it requires organizational processes through which new information modifies routines, practices, assumptions, or system properties. Recent synthesis of healthcare learning and improvement research similarly conceptualizes learning as the structured updating of organizational properties in response to new information [71].

The finding also closely aligns with the high-reliability organization tradition developed by Weick et al. [47]. High-reliability organizations do not treat failures, anomalies, and near misses as events to be administratively closed; they treat them as sources of intelligence about the functioning of the system. Collective mindfulness depends precisely on sustained attention to small failures and unexpected outcomes because these can reveal weaknesses before they produce further harm [47,48]. Healthcare applications of this framework similarly emphasize learning from errors and close calls as a foundation of reliability. Medico-legal autopsy fits unusually well within this logic because it generates evidence at the point at which the consequences of system performance are most definitive. Yet evidence alone does not constitute organizational learning. The present results suggest that perceived institutional significance is positively associated with professionals’ assessments of organizational capacity to interpret postmortem findings, integrate them into accumulated knowledge, and utilize them as lessons relevant to future practice. The critical boundary is therefore not between autopsy and no autopsy, but between autopsy as documentation and autopsy as learning.

### 5.3. Perceived System Value as an Intermediate Evaluative Construct

Perceived System Value occupies the pivotal position in the structural model. It was positively associated with Medico-Legal Risk Mitigation, Financial Risk Mitigation, and Organizational Learning Capability and showed the largest standardized direct association with Institutional Adoption Intention. Moreover, these specified predictors jointly accounted for more than half of its variance. This positioning suggests that Institutional Adoption Intention is associated not only with the recognition of diagnostic, medico-legal, or evidentiary usefulness but also with a broader evaluation of the perceived value of autopsy for the healthcare system. This mechanism resonates with Rogers’ [53] Diffusion of Innovations theory, in which perceived relative advantage is central to adoption decisions. The present study extends this reasoning to a practice that is not conventionally framed as an innovation-adoption problem. Medico-legal autopsy is not a new technology, yet its systematic institutional use requires an analogous evaluative judgment: whether its benefits are sufficiently meaningful at the system level to justify continued or expanded integration into organizational routines.

The mediation through Perceived System Value sharpens this argument. Organizational Learning Capability exhibited a significant indirect association with Institutional Adoption Intention through Perceived System Value. Perceived System Value therefore represents an intermediate evaluative construct statistically linking perceived organizational learning capability with institutional willingness to adopt medico-legal autopsy. This interpretation may provide a conceptual basis for examining differences in institutional willingness to adopt autopsy-based learning among organizations exposed to comparable medico-legal evidence.

### 5.4. Sequential Indirect Associations Linking Risk Mitigation and Institutional Adoption Intention

One of the most theoretically relevant findings is the significant sequential indirect association linking Medico-Legal Risk Mitigation to Institutional Adoption Intention through Financial Risk Mitigation and Perceived System Value. Rather than representing only separate dimensions of perceived autopsy benefits, these relationships are consistent with a theoretically specified sequence of statistical associations: medico-legal risk mitigation → financial risk mitigation → perceived system value → institutional adoption intention. This sequence represents the structure of the hypothesized model rather than an empirically established temporal or causal process.

The significance of this sequence lies in identifying a theoretically meaningful pattern of associations between different dimensions of perceived autopsy value and institutional adoption intention. Perceived medico-legal benefits are positively associated with perceived financial risk mitigation, which is positively associated with broader system-level valuation of autopsy. Perceived System Value is, in turn, positively associated with Institutional Adoption Intention. The sequential mediation analysis therefore provides statistical support for the hypothesized pattern of indirect associations, without demonstrating a temporal process of value translation or causal mediation.

This mechanism is consistent with high-reliability thinking. High-reliability organizations actively seek information contained in failures because such information can reveal vulnerabilities that remain actionable beyond the event itself [48]. The present model adds a medico-legal dimension to this logic by identifying statistical associations consistent with the potential organizational relevance of postmortem evidence for anticipating and managing future risk. Autopsy is therefore retrospective in temporal orientation but prospective in organizational function. Its potential institutional value may reside not only in explaining a death but also in the perceived relevance of postmortem findings for organizational risk recognition, learning, and prevention.

### 5.5. Theoretical Implications

The principal theoretical contribution of this study lies in changing the level at which medico-legal autopsy is conceptualized. Existing scholarship has predominantly treated autopsy at the case level, emphasizing diagnostic discrepancy, cause-of-death determination, evidentiary clarification, and forensic utility [24]. The present model introduces an organizational level of analysis, examining associations between perceived autopsy benefits and broader dimensions of risk governance, organizational learning, system valuation, and institutional decision-making. This shift enables a connection between three theoretical traditions that have largely developed separately. Reason’s [70] organizational accident perspective explains why evidence from adverse outcomes can reveal latent system vulnerabilities; high-reliability theory explains why organizations differ in their capacity to learn from such evidence [47,48]; and diffusion theory explains why recognized organizational advantage can become willingness to institutionalize a practice [53]. The present findings therefore position medico-legal autopsy at the intersection of diagnostic evaluation, organizational learning, risk governance, and institutional decision-making rather than treating it solely as a procedure for retrospective case resolution.

On this basis, we propose the concept of post-event organizational intelligence as a theoretical perspective on the potential organizational relevance of information generated through retrospective investigation of a completed adverse outcome. This perspective emphasizes the distinction between the production of postmortem evidence and its potential utilization in organizational learning, risk management, and future institutional decision-making. An autopsy may therefore be diagnostically informative without necessarily being perceived as organizationally valuable; the latter is conceptually associated with the capacity to interpret and utilize its findings in understanding and managing future risk. This distinction offers a broader theoretical explanation for the institutional relevance of medico-legal autopsy. Its value is not determined solely by what the procedure discovers, but by whether the organization possesses the capability to convert what is discovered into actionable institutional knowledge.

### 5.6. Practical and Clinical-Organizational Implications

The practical implication is that increasing the institutional contribution of medico-legal autopsy requires attention not only to whether autopsies are performed, but also to how their findings are communicated, interpreted, and incorporated into subsequent clinical and organizational learning. Hospitals should establish formal feedback pathways connecting relevant postmortem findings with morbidity and mortality reviews, diagnostic-quality programs, patient-safety committees, clinical audit, risk management, and professional education. Such pathways should enable recurrent diagnostic discrepancies, latent vulnerabilities, and potentially preventable patterns to be identified across cases rather than leaving each autopsy as an isolated investigation. This requires structured communication among forensic medicine, pathology, clinical departments, legal services, quality and safety units, and hospital management, with appropriate safeguards for confidentiality, professional accountability, and legal restrictions.

The findings also suggest that managers seeking to strengthen institutional use of autopsy should articulate its value in system-level rather than exclusively diagnostic terms. Business cases and governance discussions can incorporate its contribution to risk clarification, organizational learning, quality assurance, prevention of recurrent failures, and reduction of avoidable organizational exposure. In practical terms, the relevant managerial question is therefore not simply “How many autopsies are performed?” but “What does the organization do with what autopsies reveal?”

### 5.7. Policy and Healthcare Governance Implications

At the policy level, the findings question the adequacy of autopsy rates as the principal indicator of institutional engagement with postmortem investigation. Utilization rates capture procedural volume but not whether the knowledge generated by autopsy contributes to healthcare improvement. Governance frameworks could therefore complement conventional indicators with measures of knowledge utilization: whether relevant findings enter quality-improvement processes, whether recurrent discrepancies are systematically reviewed, and whether identified vulnerabilities result in documented organizational responses. This approach also implies a stronger interface between medico-legal systems and healthcare quality governance. Where legally and ethically permissible, aggregated and de-identified autopsy findings could contribute to institutional or system-level surveillance of diagnostic vulnerabilities, recurrent adverse patterns, and areas requiring preventive intervention. Policy should therefore distinguish between performing autopsies and learning from autopsies. Under such a governance model, medico-legal autopsy is not only the endpoint of death investigation but a potential input into a wider feedback system for healthcare quality and risk management.

### 5.8. Limitations and Future Research

Several limitations define the boundaries of these conclusions. The cross-sectional design does not permit causal inference or the establishment of temporal ordering among the constructs. Although the hypothesized associations and indirect relationships received statistical support, the proposed sequence remains theoretically specified rather than empirically verified as a temporal or causal process. Longitudinal research should examine whether changes in organizational learning capability and systematic use of autopsy findings subsequently alter perceived system value and actual institutional adoption. The outcomes also reflect professional perceptions and adoption intentions rather than objectively observed organizational behavior. Future studies should connect these constructs with institutional indicators such as autopsy utilization, frequency of autopsy-informed clinical audits, changes to diagnostic protocols, medico-legal claims, patient-safety interventions, and documented organizational responses to postmortem findings.

The multidisciplinary composition of the sample represents an important strength for examining a phenomenon spanning medicine, law, and management; however, it also introduces potential heterogeneity in respondents’ perceptions. Professional background, institutional setting, and previous direct experience with medico-legal autopsy may be associated with differences in how respondents evaluate medico-legal risk mitigation, organizational learning capability, financial risk mitigation, perceived system value, and institutional adoption intention. These characteristics were not included as covariates or examined through subgroup-specific structural analyses; consequently, the stability of the estimated structural relationships across professional backgrounds, institutional settings, and levels of direct experience cannot be established. The reported structural coefficients should therefore be interpreted as associations estimated for the overall multidisciplinary sample rather than as relationships necessarily applicable to each subgroup. Furthermore, measurement invariance across professional subgroups was not assessed, and the equivalence of the measurement structure across forensic medicine specialists, pathologists, clinicians, legal professionals, and healthcare managers cannot be assumed. Future research should employ covariate-adjusted and subgroup-specific analyses to evaluate the robustness of the observed associations, while multi-group confirmatory factor analyses could establish whether the constructs demonstrate comparable measurement properties across professional categories.

Furthermore, the study was conducted exclusively within the Serbian institutional and legal context, which limits the generalizability of the findings to other healthcare systems. Differences in medico-legal regulations, autopsy practices, healthcare financing, organizational governance, and institutional accountability may be associated with different patterns of perceived autopsy value and adoption intention across jurisdictions. Cross-national replication is therefore necessary to assess the applicability and stability of the observed associations in different institutional settings. Finally, future research should examine whether the proposed pattern of associations is predictive of actual institutional adoption, rather than adoption intention alone. Linking longitudinal changes in the proposed constructs to observable institutional practices would provide a stronger assessment of the model’s predictive validity and clarify whether stated adoption intentions are followed by corresponding organizational behavior.

## 6. Conclusions

Medico-legal autopsy occupies an unusual position in healthcare: it examines an outcome that can no longer be changed, yet the diagnostic and postmortem evidence it produces can still inform decisions affecting outcomes that have not yet occurred. This study suggests that its contemporary relevance may be evaluated not only in terms of the accuracy with which it reconstructs the past, but also in relation to the perceived capacity of healthcare organizations to utilize that knowledge in future decision-making. This perspective reframes the institutional question surrounding autopsy. The issue is not simply whether the procedure retains diagnostic value in an era of increasingly sophisticated diagnostic technologies, but how its perceived benefits are associated with organizational learning capability, risk mitigation, and institutional adoption intention. Conceptually, treating postmortem evidence as an organizational resource rather than merely the endpoint of an investigation offers a perspective that connects forensic inquiry with healthcare improvement. Information generated through postmortem investigation may have potential value for institutional memory, the recognition of otherwise overlooked vulnerabilities, and decision-making beyond the resolution of an individual case.

The broader significance of medico-legal autopsy may therefore lie in its ability to give adverse outcomes an institutional afterlife. A death investigation cannot alter what has happened, but the knowledge derived from it may provide a basis for organizational learning, greater awareness of vulnerabilities, and more informed responses to similar risks in the future. In this sense, the enduring relevance of autopsy may lie not only in the diagnostic precision with which it clarifies the past, but also in its potential to provide knowledge that healthcare organizations can draw upon in future learning, risk management, and institutional decision-making.

## Figures and Tables

**Table 1:** Exploratory Factor Analysis Results and Factor Loadings.

	Factor				
	Perceived System Value	Organizational Learning Capability	Institutional Adoption Intention	Financial Risk Mitigation	Medico-Legal Risk Mitigation
Legal Uncertainty	−0.021	−0.001	−0.039	−0.042	0.743
Liability Assessment	0.040	0.006	0.049	0.007	0.732
Dispute Resolution	0.043	0.000	0.014	0.005	0.790
Legal Security	−0.023	0.009	−0.002	0.027	0.792
Case Transparency	−0.012	0.039	−0.027	0.024	0.832
Diagnostic Discrepancies	−0.004	0.703	−0.011	−0.032	−0.012
System Weaknesses	0.024	0.738	0.004	0.009	0.011
Protocol Improvement	−0.026	0.804	−0.038	−0.001	0.030
Professional Learning	−0.019	0.846	0.009	0.058	−0.032
Problem Prevention	−0.009	0.847	−0.037	−0.004	0.011
Cost Avoidance	−0.061	0.017	−0.044	0.648	0.025
Financial Consequences	0.081	−0.030	0.034	0.667	−0.003
Risk Management	−0.013	0.007	−0.030	0.789	−0.035
Loss Prevention	0.045	0.011	0.026	0.767	0.035
Risk Assessment	−0.007	0.013	0.009	0.889	−0.034
Resource Justification	0.644	0.013	−0.054	−0.028	−0.015
Investment Value	0.635	0.056	−0.040	0.053	0.040
Benefit Advantage	0.777	−0.016	−0.013	−0.015	0.028
Resource Efficiency	0.807	−0.023	0.033	−0.010	0.010
Management Value	0.835	0.022	0.016	0.013	0.016
Risk Integration	0.026	−0.039	−0.698	−0.027	−0.028
Resource Allocation	0.023	−0.003	−0.705	−0.021	0.038
Strategic Adoption	−0.028	0.045	−0.756	0.006	0.020
Decision Integration	−0.011	0.020	−0.777	0.036	0.007
Adoption Support	0.006	0.000	−0.838	0.038	−0.012

**Table 2:** Confirmatory Factor Analysis: Composite Reliability and Convergent Validity.

Construct	Factor	CR	AVE
Perceived System Value	F1	0.890	0.618
Organizational Learning Capability	F2	0.893	0.628
Institutional Adoption Intention	F3	0.875	0.585
Financial Risk Mitigation	F4	0.872	0.578
Medico-Legal Risk Mitigation	F5	0.875	0.584

**Table 3:** Fornell–Larcker Criterion.

Construct	MLRM	OLC	IAI	FRM	PSV
Medico-Legal Risk Mitigation (MLRM)	0.764				
Organizational Learning Capability (OLC)	0.440	0.792			
Institutional Adoption Intention (IAI)	0.675	0.391	0.765		
Financial Risk Mitigation (FRM)	0.597	0.084	0.400	0.760	
Perceived System Value (PSV)	0.564	0.107	0.371	0.629	0.786

**Table 4:** Discriminant Validity: HTMT Ratios.

Construct	MLRM	OLC	IAI	FRM	PSV
Medico-Legal Risk Mitigation (MLRM)	—				
Organizational Learning Capability (OLC)	0.442	—			
Institutional Adoption Intention (IAI)	0.680	0.389	—		
Financial Risk Mitigation (FRM)	0.596	0.079	0.397	—	
Perceived System Value (PSV)	0.559	0.100	0.365	0.629	—

**Table 5:** Structural Model Results and Direct Hypothesis Testing.

Hypothesis	Structural Path	β	S.E.	C.R.	*p*	Decision
H1	Medico-Legal Risk Mitigation (MLRM) → Financial Risk Mitigation (FRM)	0.629	0.031	19.047	<0.001	Supported
H2	Medico-Legal Risk Mitigation (MLRM) → Perceived System Value (PSV)	0.284	0.028	7.546	<0.001	Supported
H3	Financial Risk Mitigation (FRM) → Perceived System Value (PSV)	0.401	0.032	10.128	<0.001	Supported
H4	Organizational Learning Capability (OLC) → Perceived System Value (PSV)	0.402	0.030	12.409	<0.001	Supported
H5	Perceived System Value (PSV) → Institutional Adoption Intention (IAI)	0.674	0.045	15.292	<0.001	Supported

Note: β = standardized regression coefficient; S.E. and C.R. are based on the corresponding unstandardized estimates. MLRM = Medico-Legal Risk Mitigation; FRM = Financial Risk Mitigation; PSV = Perceived System Value; OLC = Organizational Learning Capability; IAI = Institutional Adoption Intention.

**Table 6:** Mediation Analysis and Indirect Hypothesis Testing.

Hypothesis	Indirect Path	Indirect Effect	Boot S.E.	Lower	Upper	*p*	Decision
H6	Medico-Legal Risk Mitigation → Financial Risk Mitigation → Perceived System Value	0.191	0.019	0.155	0.230	<0.001	Supported
H7	Organizational Learning Capability → Perceived System Value → Institutional Adoption Intention	0.255	0.023	0.213	0.301	<0.001	Supported
H8	Medico-Legal Risk Mitigation → Financial Risk Mitigation → Perceived System Value → Institutional Adoption Intention	0.132	0.014	0.105	0.161	<0.001	Supported

Note: Indirect associations are represented by unstandardized user-defined estimates. Boot S.E. = bootstrap standard error; Statistical support for an indirect association is indicated when its 95% bootstrap confidence interval does not include zero. These estimates do not establish causal mediation.

**Table 7:** Explained Variance of Endogenous Constructs.

Endogenous Construct	R^2^	Explained Variance (%)
Financial Risk Mitigation	0.396	39.6%
Perceived System Value	0.546	54.6%
Institutional Adoption Intention	0.455	45.5%

## Data Availability

The original contributions presented in this study are included in the article. Further inquiries can be directed to the corresponding author.

## Appendix A

**Table A1:** Measurement Items and Final Item Retention.

Item	Statement	Item Label	Final Status
v1	Medico-legal autopsy enables more reliable determination of facts relevant to medico-legal cases.	Fact Determination	Removed
v2	Autopsy findings reduce uncertainty regarding the cause and circumstances of death.	Legal Uncertainty	Retained
v3	Autopsy findings provide an objective basis for assessing potential professional liability.	Liability Assessment	Retained
v4	Medico-legal autopsy contributes to fairer resolution of disputed medico-legal cases.	Dispute Resolution	Retained
v5	Medico-legal autopsy enhances legal security for healthcare professionals and healthcare institutions.	Legal Security	Retained
v6	Medico-legal autopsy contributes to greater transparency in cases involving uncertainty about a patient’s death.	Case Transparency	Retained
v7	Autopsy findings enable the identification of diagnostic discrepancies that were not recognized during clinical care.	Diagnostic Discrepancies	Retained
v8	Analysis of autopsy findings enables healthcare institutions to learn from previous clinical cases.	Organizational Learning	Removed
v9	Autopsy findings help identify systemic weaknesses in the delivery of healthcare.	System Weaknesses	Retained
v10	Knowledge obtained from autopsies can be used to improve clinical protocols and procedures.	Protocol Improvement	Retained
v11	Autopsy findings represent an important source of professional learning for healthcare professionals.	Professional Learning	Retained
v12	Systematic analysis of autopsy findings can help prevent similar problems from recurring in future cases.	Problem Prevention	Retained
v13	Reliable determination of facts through autopsy can reduce financial uncertainty associated with medico-legal cases.	Financial Uncertainty	Removed
v14	Autopsy findings can help avoid unnecessary costs associated with prolonged legal and administrative proceedings.	Cost Avoidance	Retained
v15	Medico-legal autopsy can help limit the financial consequences of unclear or disputed deaths.	Financial Consequences	Retained
v16	Information obtained through autopsy can contribute to better management of financial risks within healthcare institutions.	Risk Management	Retained
v17	The use of autopsy findings in risk management can help prevent future financial losses.	Loss Prevention	Retained
v18	Autopsy findings can help healthcare institutions better assess the financial consequences of medico-legal risks.	Risk Assessment	Retained
v19	The benefits of medico-legal autopsy to the healthcare system justify the resources required for its performance.	Resource Justification	Retained
v20	Information obtained through medico-legal autopsy creates substantial value for the healthcare system.	System Value	Removed
v21	Medico-legal autopsy represents a justified investment of resources when its long-term benefits are considered.	Investment Value	Retained
v22	The benefits of medico-legal autopsy outweigh its immediate costs.	Benefit Advantage	Retained
v23	Systematic use of autopsy findings can contribute to more efficient use of healthcare system resources.	Resource Efficiency	Retained
v24	Medico-legal autopsy represents a valuable instrument for risk management within the healthcare system.	Management Value	Retained
v25	Healthcare institutions should systematically integrate medico-legal autopsy findings into their risk management processes.	Risk Integration	Retained
v26	Autopsy findings should be routinely used to improve the quality of healthcare institutions.	Quality Integration	Removed
v27	Healthcare systems should allocate adequate resources to develop capacity for medico-legal autopsies.	Resource Allocation	Retained
v28	Medico-legal autopsy should play a greater role in institutional risk management strategies.	Strategic Adoption	Retained
v29	Medico-legal autopsy findings should be systematically incorporated into decisions concerning healthcare safety and quality.	Decision Integration	Retained
v30	I would support broader institutional adoption of medico-legal autopsy as a healthcare risk management tool.	Adoption Support	Retained

## Appendix B

**Table A2:** Full-Collinearity VIF Assessment of Common Method Bias.

Construct	N	R^2^	Full-Collinearity VIF	Threshold	Result
Medico-Legal Risk Mitigation (MLRM)	1035	0.542	2.183	3.3	Acceptable
Organizational Learning Capability (OLC)	1035	0.203	1.255	3.3	Acceptable
Institutional Adoption Intention (IAI)	1035	0.373	1.595	3.3	Acceptable
Financial Risk Mitigation (FRM)	1035	0.398	1.661	3.3	Acceptable
Perceived System Value (PSV)	1035	0.367	1.580	3.3	Acceptable

Note: Full-collinearity VIF values were calculated as 1/(1 − R^2^), with each composite construct score regressed on the remaining four composite scores. The threshold of 3.3 follows [69]. Values below this threshold indicate no problematic full collinearity but do not definitively exclude common method bias.
